# Data of de novo assembly of fruit transcriptome in *Aegle marmelos* L.

**DOI:** 10.1016/j.dib.2019.104189

**Published:** 2019-06-24

**Authors:** Prashant Kaushik, Shashi Kumar

**Affiliations:** a Instituto de Conservación y Mejora de la Agrodiversidad Valenciana, Universitat Politècnica de València, Valencia, Spain; b International Center for Genetic Engineering and Biotechnology, Aruna Asaf Ali Marg, New Delhi 110 067, India

**Keywords:** *De novo* assembly, Transcriptome, Bael, Fruit, *Aegle marmelos*

## Abstract

*Aegle marmelos* L. (Bael), of family Rutaceae, produces nutritious and medicinally important fruits. Here, we provide the first information regarding the de novo transcriptome assembly of *Aegle marmelos* L. fruit. The information on the fruit transcriptome sequencing data will be useful to gain a better insight into the important pathways in the *Aegle marmelos* L. fruits. The data can be accessed via NCBI BioProject (id PRJNA433585).

**Table:** Specifications table

Subject area	*Plant Biology*
More specific subject area	*Transcriptomics*
Type of data	*Assembly of reads and sequence annotation, table, figure*
How data was acquired	*cDNA sequencing was performed using Illumina HiSeq 2500*
Data format	*Raw (FASTQ) sequences*
Experimental factors	*Fruit flesh tissues at commercial ripe stage*
Experimental features	*Commercially ripe fruits of Aegle marmelos* L*. cultivar “Kaghzi” were used for RNA extraction. Paired-end reads were generated using the HiSeq 2500 system. Further, pair-end reads were assembled using Trinity.*
Data source location	*Kurukshetra, India*
Data accessibility	*Accessible as NCBI BioProject (PRJNA433585).*https://www.ncbi.nlm.nih.gov/sra/SRX5010282[accn]

**Table:** 

**Value of the data** • To our knowledge, this is the first dataset regarding the fruit transcriptome in *A. marmelos*. • Data will be useful for the genetic improvement of *A. marmelos* fruits. • Further, this information can be used for the identification of metabolic pathways present in the *A. marmelos* fruit.

## 1 Data

**Fig. 1:**
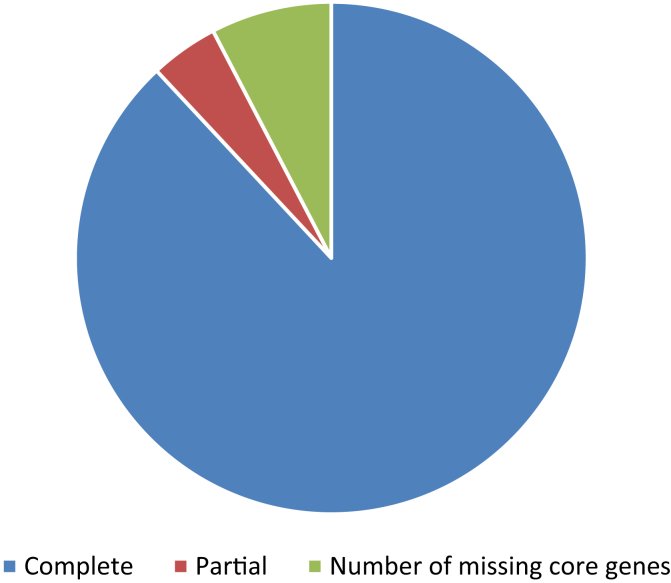
Representation of completeness scores of *A. marmelos* fruit transcriptome assembly.

Recently, we have published the first report of transcriptome data of leaf transcriptome in *A. marmelos* [1], [2]. But, there is no information regarding the fruit transcriptome data of *A. marmelos*. Here, we provide useful information regarding fruit transcriptomic data generated using Illumina HiSeq 2500 platform. The reads were de novo assembled using the Trinity software program. After that, length statistics, and the overall composition of bael fruit transcriptome assembly was determined. Finally, we determined the completeness scores of *A. marmelos* fruit transcriptome assembly. Table 1 particulars the information of statistics and composition of the bael fruit transcriptome assembly. Finally, Table 2 and Fig. 1 provides the information regarding the completeness scores of *A. marmelos* fruit transcriptome assembly.

**Table 1:** Length statistics and composition of bael fruit transcriptome assembly.

Parameters	Statistics
Number of sequences	82,656
Total length (nt)	92,669,040
Longest sequence (nt)	16,700
Shortest sequence (nt)	201
Mean sequence length (nt)	1121
Median sequence length (nt)	652
N50 sequence length (nt)	1953
L50 sequence count	15,317
Number of sequences > 1K (nt)	32,565
Number of sequences > 10K (nt)	28
Base composition (%)	A: 29.77
	T: 29.69
	G: 20.37
	C: 20.17
GC-content (%)	40.54

**Table 2:** Completeness assessment report.

Parameters	Statistics
Total number of core genes queried	1440
Number of core genes detected	
Complete	1268 (88.06%)
Complete + Partial	1330 (92.36%)
Number of missing core genes	110 (7.64%)
Average number of orthologs per core genes	1.66
% of detected core genes that have more than 1 ortholog	46.14

## 2 Experimental design, materials, and methods

Fresh fruits at the commercially ripe (i.e. physiologically immature) stage were harvested from three healthy plants of round fruit shaped variety “Kagzi” growing at the Government Garden Nursery, Kurukshetra, India. The trees were approximately 5-year-old, and fruit sampling was performed in August 2018. The RNA was extracted from the fresh fruit samples using the.

RNeasy Mini Kit (Qiagen) following the manufacturer's instructions. Extracted RNA of the three carefully chosen fruits were pooled in the equimolar concentration to constitute one sample for cDNA library preparation using the TruSeq RNA Library Prep Kit v2 (Illumina, Inc., USA). The RNA was quantified by Agilent 2100 Bioanalyzer (Agilent, USA) and also on the 1% agarose gel. Further, paired-end reads were generated using HiSeq 2500 (2 × 150 bp chemistry) system. The raw reads were filtered using Trimmomatic ver. 0.36 to get rid of adapters and low-quality reads (Phred score ˂ 20) [3].

A total of raw reads 49.58 million raw reads were further cleaned to 47.71 million good quality reads. These good quality reads were assembled de novo using the Trinity software package (version 2.4.0), with a minimum read length of 200 and K-mer size of 25 [4]. A total of 82,656 sequences were assembled with a GC-content of 40.54% (Table 1). We further determined the completeness of our transcriptome assembly using the Bench-marking universal single-copy orthologs (BUSCO) software version 3 in the recently developed web-based server gVolante [5]. Out of the total 1440 genes enquired 1268 were completely present (Fig. 1). With more than one ortholog present determined for 46.14% of genes that represented around 1.66 orthologs per core gene in our transcriptome assessment report.
